# Unbalanced Occlusion Modifies the Pattern of Brain Activity During Execution of a Finger to Thumb Motor Task

**DOI:** 10.3389/fnins.2019.00499

**Published:** 2019-05-17

**Authors:** Maria Paola Tramonti Fantozzi, Stefano Diciotti, Carlo Tessa, Barbara Castagna, Daniele Chiesa, Massimo Barresi, Giulio Ravenna, Ugo Faraguna, Claudio Vignali, Vincenzo De Cicco, Diego Manzoni

**Affiliations:** ^1^Department of Translational Research and of New Surgical and Medical Technologies, University of Pisa, Pisa, Italy; ^2^Department of Electrical, Electronic and Information Engineering “Guglielmo Marconi,” University of Bologna, Cesena, Italy; ^3^Department of Radiology, Versilia Hospital, Azienda USL Toscana Nord Ovest, Camaiore, Italy; ^4^Orthopedics Unit, St. Barth Hospital, Sarzana, Italy; ^5^Department of Orthopedics, University of Genoa, Genoa, Italy; ^6^Institut des Maladies Neurodégénératives, Université de Bordeaux, Bordeaux, France; ^7^Department of Developmental Neuroscience, IRCCS Fondazione Stella Maris, Pisa, Italy

**Keywords:** malocclusion, trigeminal input, bite, fingers movement, brain activation, fMRI, BOLD signal

## Abstract

In order to assess possible influences of occlusion on motor performance, we studied by functional magnetic resonance imaging (fMRI) the changes in the blood oxygenation level dependent (BOLD) signal induced at brain level by a finger to thumb motor task in a population of subjects characterized by an asymmetric activation of jaw muscles during clenching (malocclusion). In these subjects, appropriate occlusal correction by an oral orthotic (bite) reduced the masticatory asymmetry. The finger to thumb task was performed while the subject’s dental arches were touching, in two conditions: (a) with the teeth in direct contact (Bite OFF) and (b) with the bite interposed between the arches (Bite ON). Both conditions required only a very slight activation of masticatory muscles. Maps of the BOLD signal recorded during the movement were contrasted with the resting condition (activation maps). Between conditions comparison of the activation maps (Bite OFF/Bite ON) showed that, in Bite OFF, the BOLD signal was significantly higher in the trigeminal sensorimotor region, the premotor cortex, the cerebellum, the inferior temporal and occipital cortex, the calcarine cortex, the precuneus on both sides, as well as in the right posterior cingulate cortex. These data are consistent with the hypothesis that malocclusion makes movement performance more difficult, leading to a stronger activation of (a) sensorimotor areas not dealing with the control of the involved body part, (b) regions planning the motor sequence, and (c) the cerebellum, which is essential in motor coordination. Moreover, the findings of a higher activation of temporo-occipital cortex and precuneus/cingulus, respectively, suggest that, during malocclusion, the movement occurs with an increased visual imagery activity, and requires a stronger attentive effort.

## Introduction

There is a debate as to whether trigeminal sensorimotor signals may affect posture and movement. In particular, postural control seems to be influenced by the occlusal condition (Ohlendorf et al., 2014; Julià-Sánchez et al., 2015; see, however, Perinetti, 2006), temporomandibular joint (TMJ) disorders (Chaves et al., 2014) and orofacial motor activity (Kushiro and Goto, 2011; Ringhof et al., 2015). These data are consistent with the observation that sensorimotor signals elicited during isometric clenching are very effective in increasing the excitability of spinal motoneurons (Miyahara et al., 1996). On the other hand, correction of TMJ disorders by splints does not seem to affect motor performance (McArdle et al., 1984), while there is evidence that changes in the occlusal condition may affect locomotion (Okubo et al., 2010; Maurer et al., 2015) and the associated postural stabilization in normal subjects (Ohlendorf et al., 2014), as well as the overall motility in Parkinsonian patients (Nomoto et al., 2013). Finally, recent evidence indicates that in subjects showing an unbalance in sensorimotor orofacial activity (consisting in an asymmetric activation of left and right masseter muscles during clenching) the reestablishment of a symmetric condition by bite wearing enhances the speed of execution in a complex sensorimotor task (De Cicco et al., 2016).

If the occlusal condition does affect body movements, it is reasonable to assume that it will also modify also the movement-elicited local changes in cerebral blood. So far, the effects of occlusal condition on the movement-elicited changes in cerebral blood flow have been investigated only for orofacial movements. In particular, wearing a splint during jaw tapping movements decreased bilaterally the blood oxygenation level dependent (BOLD) signal in the primary and secondary sensorimotor areas, putamen, inferior parietal/prefontal cortex and anterior insula, thus indicating a decrease in the blood flow to these regions during the task. At variance, the BOLD signal increased in the prefrontal, temporal, parietal, and occipital lobes, as well as in the cerebellar network (Lotze et al., 2012).

The purpose of the present investigation was to document whether correction of an unbalanced occlusal condition may modify the pattern of brain activation induced by finger movements. For this purpose, we have analyzed, in a group of subjects showing an unbalance in sensorimotor orofacial activity during clenching, the changes in the BOLD signal elicited by a typical finger to thumb task (Shibasaki et al., 1993) and their modulation by occlusal correction.

## Materials and Methods

### Subjects

Eight subjects (4 females) of age between 49 and 69 were enrolled in the study. At maximal jaw opening, clicking sounds were present in all the subjects and two of them were also reporting pain. In two subjects, clicks appeared also at the beginning of jaw lowering. None of them was affected by neurological or psychiatric disorders. Subjects were asked to avoid caffeine containing drinks and smoking at least 2 h prior the experimental session. The study was carried out in accordance with the recommendations of the Ethical Committee of the University of Pisa. According to the Declaration of Helsinki, each subject signed an informed consent, approved by the local Ethical Committee.

### MR Acquisition Protocol

All MR exams were performed using a 1.5T MR system (MAGNETOM Avanto, Siemens Healthcare, Erlangen, Germany) equipped with 45 mT/m gradients and a quadrature head coil.

The examination protocol included (a) axial high resolution contiguous 3D T1-weighted images that were obtained with a MPRAGE sequence [repetition time (TR) = 1.900 ms, echo time (TE) = 3.4 ms, inversion time (TI) = 1100 ms, flip angle = 15°, slice thickness = 0.9 mm, field of view (FOV) = 256 mm × 256 mm, matrix size = 256 × 256, number of excitations (NEX) = 2] and (b) axial T2-weighted images that were obtained with a fluid attenuated inversion recovery (FLAIR) sequence (TR = 9.000 ms, TE = 88 ms, TI = 2.500 ms, slice thickness = 3 mm, FOV = 230 mm × 172.5 mm, matrix size = 256 × 154, turbo factor = 16, NEX = 1). Functional images were collected using a T2^∗^- weighted echo-planar imaging (EPI) sequence (TR = 3000 ms, TE = 50 ms, FA = 90, slice thickness = 4 mm; FOV = 256 mm × 256 mm, matrix size 64 × 64; number of slices = 37). The slices were oriented along and parallel to the bi-commissural plane and centered to cover the entire brain.

The task consisted of a self-paced continuous finger to thumb task, performed with the right hand, without visual feedback, that the subjects, laying supine within the scanner, repeated before data acquisition until they were able to perform correctly. The subjects were visually monitored throughout the scanning time and no gross change in performance could be observed.

The functional magnetic resonance imaging (fMRI) sequences were acquired while the subjects performed the task while their dental arches were touching in two different conditions: (a) with the teeth in direct contact (Bite OFF) and (b) with an orthotic (bite) interposed between the arches (Bite ON). Both conditions required only a very slight activation of masticatory muscles. A block design was used, where four blocks of finger to thumb task (T) were alternated with four blocks of rest (R) according to the sequence RTRTRTRT, for each condition. Start and end of each block were instructed by delivering “go” and “stop” auditory signals. Each block, in which 10 scans were acquired, lasted 30 s, for a total of 80 scans/run in 4 min. Before each run, two dummy volumes were acquired and discarded from analysis to avoid T1-related relaxation effects. The first condition tested (Bite OFF or Bite ON) was changed from subject to subject in pseudorandom order. In order to minimize head movements, head pads and forehead straps were used. The subjects were visually monitored throughout the scanning time also for non-task related movements.

### fMRI Data Analysis

Functional data were analyzed using FSL 4.1.4 software [Oxford Center for Functional Magnetic Resonance Imaging of the Brain (FMRIB) software library^1^], one of the two major and most widely employed neuroimaging analysis tools (Jenkinson et al., 2012). The following pre-statistics processing were applied to the 80 fMRI scans: motion correction using MCFLIRT (Jenkinson et al., 2002), non-brain removal using BET (Smith, 2002), spatial smoothing using a 8 mm FWHM Gaussian kernel, grand-mean intensity normalization of the entire 4D data set by a single multiplicative factor and high-pass temporal filtering. For each subject, the mean (across voxels) voxel absolute displacement (each time point with respect to the reference image, i.e., to the middle-time point rsfMRI image) calculated by MCFLIRT was less than 1 mm (equal to 1/4 the voxel dimension) and, for this reason, none of the subjects was excluded for excessive head motion. The high resolution T1-weighted images were co-registered in a standard space [Montreal Neurological Institute (MNI) 152 Brain]. This allowed assessment of activation areas in terms of the MNI coordinate system. Registration of EPI functional images to the individual high resolution T1-weighted image and standard space was carried out using affine transformation with 12 degrees of freedom (Jenkinson and Smith, 2001). Registration from high resolution structural to standard space was then further refined using FNIRT non-linear registration (Andersson et al., 2007a,b). The FMRIB’s improved linear model (Woolrich et al., 2001) was adopted for statistical analysis in order to determine the activation maps of signal changes between active vs. rest periods. Single-subject cluster analysis was performed on voxels having Z (Gaussianised T) > 3.1 and a (cluster-based corrected) significance threshold of *p* = 0.05 (Worsley, 2001). In each Bite ON and the Bite OFF condition, a within-group analysis was carried out using one-sample *t*-test to assess the differences between the active and passive blocks and a fixed effects model using FLAME (FMRIB’s Local Analysis of Mixed Effects) (Beckmann et al., 2003; Woolrich et al., 2004) by forcing the random effects variance to zero (Woolrich, 2008). The Z-statistical maps derived from the within-group analyses underwent a cluster thresholding with a Z threshold of 3.1 and a cluster p threshold of 0.05.

To assess possible differences in the BOLD activation pattern between the Bite ON and Bite OFF condition, a between-conditions analysis was carried out using paired *t*-test and a fixed effects model. Z (Gaussianised T/F) statistic images were thresholded using clusters determined by Z > 3.1 and a (corrected) cluster significance threshold of *p* = 0.05. We inserted the subject’s age as covariate variable in both within-group and within-condition models.

The coordinates of the local activation maxima observed in the present study within the clusters with a significant condition effect were inserted in the Brain Map Sleuth^2^ and in the NeuroSynth Database^3^, so to retrieve similarly localized activations reported in previously studies during different sensorimotor and cognitive tasks. The Yale Brain Map^4^ and the automated anatomical labeling (AAL) atlas (Tzourio-Mazoyer et al., 2002) were utilized in order to define the position of the local activation maxima within the Brodmann areas (BA) and within the different cortical/subcortical/cerebellar regions, respectively.

### Electromyographic Evaluation and Cusp Bite Manufacturing

**FIGURE 1:**
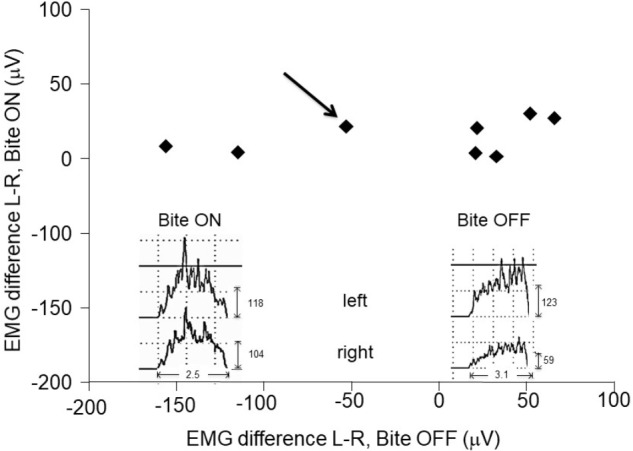
Asymmetries in EMG activity: effects of the bite. Scatter plot of left-right differences observed in masseter in EMG activity during clenching with (Bite ON, in ordinates) and without (Bite OFF, in abscissas) occlusal correction by bite wearing. The insets represent the EMG raw data recorded in a representative subject (indicated by the black arrow). Note the narrow range of the EMG asymmetry following occlusal correction.

The evaluation of the electromyographic (EMG) activity of jaw muscles during clenching was performed in a separate session, before fMRI acquisition. Only subjects with an asymmetry in EMG activity [200(EMG_left_-EMG_right_)/(EMG_left_+EMG_right_)] higher than 15% were enrolled in the study. They were submitted to a transcutaneous electrical nerve stimulation (TENS) of trigeminal motor branches (Noaham and Kumbang, 2008) for 15 min. Jaw muscles stimulation was made by means of four couples (cathode/anode) of electrodes (1600 mm^2^ of surface) positioned on both side at the level of incisura sigmoidea and of the submental region. Repeated contractions of masseters and mandible depressor muscles were obtained by means of biphasic (cathodal/anodal) current pulses (0.54 ms duration, 21–25 mA intensity), delivered by two I.A.C.E.R. stimulators (Martellago, Venice, Italy). The intensity of the left and right stimuli was adjusted in order to obtain a symmetric muscle activation (evaluated by EMG recording), while the frequency corresponded to 40 and to 0.618 Hz for mandible depressor and elevator muscles, respectively. These patterns led to an alternated contraction/relaxation of masseters and to a tonic contraction of depressor muscles, resulting in small amplitude mandibular movements (1 mm). Following TENS, the mandibular resting posture was lowered and a dental impression was obtained in the new relative position of dental arches by placing a self-hardening material between them. This dental impression was used to manufacture a cusp bite (Dao et al., 1994) modeled on the inferior dental arch. Cusp bite placement reduced the myoelectric asymmetry observed during clenching (Figure 1), which decreased to less than 15% in all the subjects.

Comparison of EMG activities during clenching in Bite ON and Bite OFF was performed by paired *t*-test.

## Results

### EMG Asymmetries

During biting with normal (uncorrected) occlusion (Bite OFF), all subjects showed an evident left-right asymmetry in the activity of the masseter (see Figure 1), whose absolute value corresponded to 64.3 ± 49.2 μV (mean ± SD of the difference between the two sides). As shown in Figure 1, wearing a cusp bite (Bite ON) reduced the asymmetry in EMG activity, which reached, on the average, the value of 12.3 ± 4.13 μV (paired *t*-test, *p* < 0.022). The reduction in the EMG asymmetry was due to a significant increase in the activity of the hypotonic masseter muscle, which raised from 35.8 ± 27.1 to 70.1 ± 23.6 μV (paired *t*-test, *p* < 0.009) joined to a non-significant activity decrease on the hypertonic side (from 100.0 ± 56 to 73.1 ± 30.1 μV: paired *t*-test, *p* < 0.101).

### Comparison Between Bite ON and Bite OFF Conditions

**FIGURE 2:**
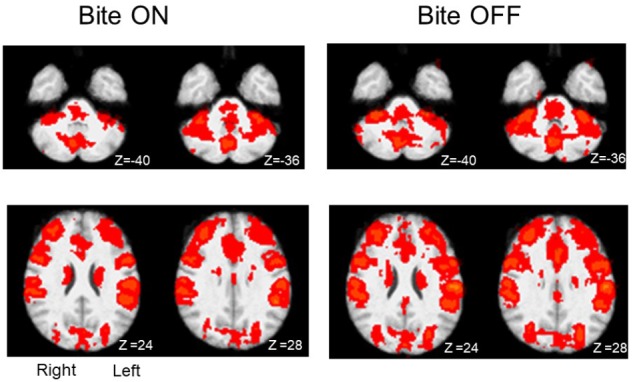
Activation maps (task-rest) obtained during finger to thumb task in Bite ON and Bite OFF conditions. The red areas indicate regions characterized by significant activation during finger to thumb task in Bite ON and Bite OFF conditions. Right and left side are indicated in the section at Z 24, in the Bite ON column (the images are depicted from a bottom view). Upper row: cerebellum. Lower row: regions around the central sulcus. Z coordinates in mm in the MNI standard space are indicated under each section.

In both Bite OFF and Bite ON conditions, the finger to thumb task modified significantly the BOLD signal with respect to rest in several cortical and subcortical structures. As shown in Figure 2, within the different regions recruited by the task the extent of activated voxels was larger in Bite OFF with respect to Bite ON.

**FIGURE 3:**
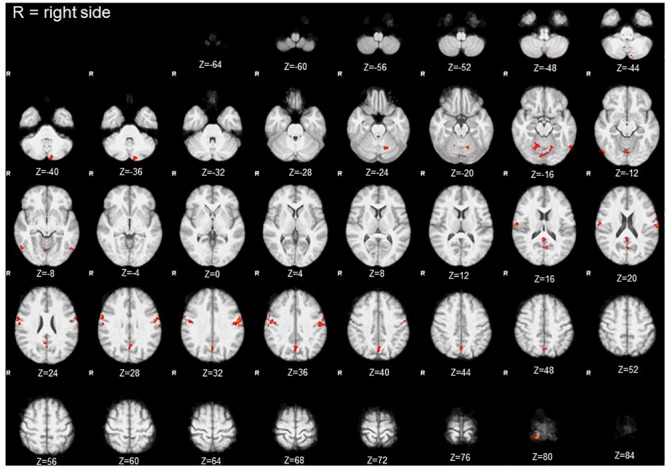
Clusters with significant condition effect (Bite OFF>Bite ON). Red areas indicate clusters with significant differences between the activations maps (task-rest) obtained in Bite OFF and Bite ON conditions during finger to thumb task. The letter R indicates the right side (the images are depicted from a bottom view). Z coordinates in mm in the MNI standard space are indicated under each section.

Comparison of activation maps (task minus resting condition) obtained in Bite OFF and Bite ON indicated the presence of clusters of voxels showing a significantly higher task-related activation in Bite OFF, while the opposite behavior has never been observed. All these clusters are shown in Figure 3, while the MNI coordinates of the corresponding centers of gravity (GOGs) are indicated in Table 1, together with the number of significant voxels. Data about the localization of the local activation maxima are given in Table 2 for each of the significant clusters. Three clusters (two on the right and one on the left side) were located in the lower part of the precentral gyrus, in an area corresponding to the trigeminal sensorimotor region (Penfield and Boldrey, 1937). An additional cluster lied within the post central gyrus, at the level of the body somatotopic area above the hand region. In the precentral gyrus, the clusters included BA 4 and 6, while postcentral activation interested the primary somatosensory region and BA 40 on the right side.

**Table 1:** Cluster of voxels showing a significant condition effect (Bite OFF>Bite ON).

Number	Activated volume (voxels)	Z Max	X (mm)	Y (mm)	Z (mm)	Localization of the center of gravity	Side
1	296	4.88	–58	–9	30	Postcentral gyrus	Left
2	171	5	61	–7	25	Postcentral gyrus	Right
3	91	5.85	18	–44	81	Postcentral gyrus	Right
4	71	4.46	52	–8	36	Postcentral gyrus	Left
5	149	4.92	–5	–70	–17	Cerebellum lobule VI,	Left
6	93	4.52	–10	–86	–39	Cerebellum Crus II	Left
7	53	4.36	18	–61	–17	Cerebellum lobule VI	Right
8	74	4.41	–57	–65	–13	Occipital Inferior gyrus	Right
9	62	4.94	57	–70	–11	Temporal Inferior gyrus	Left
10	275	4.94	0	–61	29	Precuneus	Left

*The localization (according to the AAL atlas) of clusters of voxels showing a significant condition effect is given together with the Z Max value and the MNI coordinates of their center of gravity.*

**Table 2:** Coordinates and localization of local activation maxima observed within each of the ten clusters showing a significant condition effect.

Cluster	Z Max	Local maxima x (mm)	Local maxima y (mm)	Local maxima z (mm)	Localization	Side
1	4.88	–66	–16	20	Postcentral gyrus	Left
	4.82	–56	–6	30	Precentral gyrus	Left
	4.63	–64	–2	22	Postcentral gyrus	Left
	4.31	–62	–16	18	Postcentral gyrus	Left
	4.28	–56	–16	36	Postcentral gyrus	Left
	4.03	–52	–16	36	Postcentral gyrus	Left
2	5	64	2	28	Precentral gyrus	Right
	4.84	62	–14	16	Postcentral gyrus	Right
	4.65	62	–12	22	Postcentral gyrus	Right
	4.03	58	4	38	Precentral gyrus	Right
	3.96	60	–12	30	Postcentral gyrus	Right
	3.93	60	–14	36	Postcentral gyrus	Right
3	5.84	14	–42	80	Postcentral gyrus	Right
	3.71	26	–44	76	Postcentral gyrus	Right
4	4.46	52	–8	36	Postcentral gyrus	Right
	3.71	46	–10	36	Postcentral gyrus	Right
	3.59	56	–8	42	Precentral gyrus	Right
	3.49	60	–4	38	Postcentral gyrus	Right
5	4.92	4	–72	–10	Vermis lobule VI	–
	4.36	–14	–62	–20	Cerebellum lobule VI	Left
	4.36	–2	–78	–18	Vermis lobule VII	–
	4.24	6	–82	–18	Cerebellum Crus I	Right
	4.1	2	–80	–16	Vermis lobule VI	–
	3.64	–2	–74	–12	Vermis lobule VI	–
6	4.51	–12	–86	–36	Cerebellum Crus II	Left
	3.58	–12	–86	–48	Cerebellum Crus II	Left
7	4.36	20	–64	–16	Cerebellum lobule VI	Right
	4.22	16	–58	–16	Cerebellum lobule VI	Right
8	4.41	–54	–66	–8	Temporal inferior gyrus	Left
	4.06	–60	–66	–14	Occipital inferior gyrus	Left
9	4.94	58	–70	–12	Temporal inferior gyrus	Right
	4.02	52	–74	–12	Occipital Inferior gyrus	Right
	3.94	56	–60	–8	Temporal Inferior gyrus	Right
10	4.94	2	–46	18	Precuneus	Right
	4.3	2	–62	14	Calcarine	Right
	4.07	2	–70	34	Precuneus	Right
	4.03	0	–72	40	Precuneus	–
	3.85	–6	–64	16	Calcarine	Left
	3.81	0	–66	44	Precuneus	–

*The localization (according to the AAL atlas) and the MNI coordinates of local activation maxima included in each of the clusters of voxels with a significant condition effect is given together with the corresponding Z Max value.*

Within the cerebellum, three clusters were observed. One of them was centered in paravermal region of the lobule VI on the right side. The second one, was limited to Crus II in the left hemisphere. Finally, the third cluster encompassed the vermal cortex of lobule VI and, to some extent of lobule VII, as well as the paravermal cortex of Crus I on the right side and that of lobule VI on the left.

Finally, two roughly symmetrical clusters could be found at the level of right and left inferior temporal/occipital lobes, while, caudally on the inner brain surface, a cluster of significant voxel extended bilaterally from the calcarine cortex down to the precuneus, expanding in the posterior cingulate region of the right side.

## Discussion

### Difference in BOLD Signal Between Bite OFF and Bite ON: General Considerations

The present data indicate that the increase in cerebral blood flow elicited by a motor task involving the fingers can be influenced by the occlusal condition. In fact, when subjects wore a bite that balanced the occlusion, the increase in BOLD signal elicited by finger movements was significantly reduced at the level of frontoparietal sensorimotor regions, cerebellum, temporo-occipital, and midline posterior regions.

So far the effect of malocclusion was tested only on jaw-related brain activation, which was enhanced (Lotze et al., 2012), similarly to what observed for hand movements-related activation in the present study. It is known that cortical activation decreases with increasing skill and automaticity (see Saling and Phillips, 2007, for ref) of the performed movement, probably due to a higher coupling between sensorimotor regions (Wu et al., 2008). So, a higher Bite OFF activation indicates that malocclusion may be detrimental to motor performance, imposing a higher attentive effort (see Wu et al., 2008) and, possibly, leading to a decoupling of the areas involved in the task.

### Fronto-Parietal Sensorimotor Regions

As shown in Table 2, three clusters (1,2,4) were located in the fronto-parietal sensorimotor regions of both sides. They largely overlapped with the trigeminal regions of the primary motor and somatosensory cortex, which have been defined by stimulation experiments in humans (Penfield and Boldrey, 1937) and show activation during different types of orofacial sensorimotor and speaking tasks (Watanabe et al., 2004; Grabski et al., 2012; Iida et al., 2012; Lotze et al., 2012; Quintero et al., 2013). Since the cortical activation elicited by movement of a given body part spreads outside of the corresponding sensorimotor representation (Stippich et al., 2007), the larger activation of the sensorimotor trigeminal region in Bite OFF could be to a less selective recruitment of somatotopic map and/or to an increased recruitment of inhibitory interneurons which suppress the output of the regions inappropriate for the motor task in execution. In the former case, the higher activation in Bite OFF observed within the primary somatosensory cortex could be due to (a) a stronger orofacial input elicited by activation of masticatory muscles during finger movements and/or (b) to a stronger reactivation by efference copies from the trigeminal motor region (Cui et al., 2014).

Sensorimotor clusters extend from the primary motor region (BA 4) to BA 6 that, in monkey, controls not only orofacial, but also finger movements (Rizzolatti et al., 1988). If this region has the same function in monkeys and humans, its higher activation in Bite OFF could reflect a more difficult planning of the finger to thumb sequence. Indeed, fMRI experiments in humans have shown that regions overlapping with the significant clusters in BA 6 are activated during planning and execution of finger and hands movements (Jankowski et al., 2009; Plata Bello et al., 2015).

Postcentral clusters extend into the right parietal associative cortex (area 40), whose lesion impairs language function in humans (Sakurai et al., 2010). However, networks located in the corresponding areas of the brain monkey are involved in the control of finger movements (Vingerhoets, 2014). If this is the case also in humans, the higher activation of these regions in Bite OFF condition could reflect a higher attentive cost and a reduced skill of the performed finger movements (see Saling and Phillips, 2007). This hypothesis is in agreement with the fact that, in humans, these areas are activated not only during orofacial (Watanabe et al., 2004; Quintero et al., 2013) but also during hand sensorimotor activities, such as imagery and execution of hand movements (de Vries et al., 2009) and active tactile discrimination (Stoeckel et al., 2003).

A fourth cluster (cluster 2, see Table 1) was confined to the trunk-arm region (Penfield and Boldrey, 1937) of the primary somatosensory cortex and to the neighboring BA 5 and 7. Its higher activation in Bite OFF could reflect a higher feedback from the trunk and arm during finger movements, possibly due a less focalized postural adjustment (Caronni and Cavallari, 2009).

### Cerebellar Structures

Within the cerebellum a significant cluster included the vermal cortex of lobules VI and VII, together with the paravermal cortex of right lobule VI and left Crus I. A second cluster was formed by left Crus II and a third one by the paravermal cortex of the right lobule VI. It is known that in humans, lobule VI is connected with the motor and premotor cortical regions, lobule VII with parietal and prefrontal regions while Crus I and Crus II with the lateral prefrontal cortex (Stoodley and Schmahmann, 2018). In all these regions, during finger to thumb task, the Bite OFF condition requires a higher cerebellar involvement for correctly driving fronto-parietal circuits. Since the cerebellum is particularly involved in motor coordination (Manto and Bastian, 2007), this increased activation is in agreement with the hypothesis that movement performance deteriorates in Bite OFF imposing an heavier computational burden on cerebellar circuits (Wu et al., 2008).

fMRI studies indicate that these cerebellar areas are activated during orofacial sensorimotor activity (Eldeghaidy et al., 2011; Thürling et al., 2011; Wong et al., 2011; Grabski et al., 2012; Quintero et al., 2013; Shoi et al., 2014), hand movements planning and execution (Jankowski et al., 2009; Callan et al., 2012), as well as during sensory and cognitive performances that may take place during the finger to thumb task, with particular reference to its spatial and temporal aspects (Diedrichsen et al., 2006; Majerus et al., 2007; O’Reilly et al., 2008; Grahn and McAuley, 2009; Mullally and Maguire, 2011; Brown and Stern, 2014; Onuki et al., 2015).

In summary, the higher cerebellar activation observed during Bite OFF can be the expression of the need of a higher coordinative and planning effort; moreover, some of the trigeminal related regions such as left and right lobule VI may be sensitive to the higher activity of the trigeminal sensorimotor cortical regions to which they are coupled.

### Temporooccipital Regions

As shown in Table 2, two significant clusters were located at the inferior temporal/occipital cortex of both sides. These areas belong to the “ventral streaming” regions, where neuronal activation leads to recognition of the seen objects and persons (Goodale, 1998). fMRI studies are consistent with these findings (Ardila et al., 2016). On these basis, it could be proposed that, during a finger to thumb task performed in Bite OFF, subjects have to produce a larger imagery effort in order to perform the movement, leading to a higher activation of structures involved in visual image recognition. According to this hypothesis, significant clusters described in the present study are activated during visual imagery (Nakao et al., 2011), visual memory load (Rahm et al., 2014), but also during visuospatial processing (Grabner et al., 2009; King and Miller, 2014), hand movement (Macaluso et al., 2007), trajectory prediction (Olsson and Lundström, 2013) and discomfort perceptions (Ogino et al., 2007). Finally, on the left side, activity has been observed also during clenching (Wong et al., 2011). All these activations might be justified by recruitment of circuits related to the visual recognition of actual/imaged objects and body parts during specific behaviors, such as the finger to thumb task.

### Midline and Limbic Structures

Significant clusters were found bilaterally on the inner faces of the hemispheres, from the calcarine cortex to the precuneus, with an extension into the right posterior cingulate gyrus. The calcarine cortex represents visual processing area (Ffytche and Catani, 2005) and its larger activation during Bite OFF suggests that, in this condition, a higher imaginative effort occurs during finger movements. The precuneus (Cavanna and Trimble, 2006) is one of the brain regions more active at rest and contribute to the so-called “default mode of brain function” (DMF) (Raichle et al., 2001): these structures are hypoactive in conditions of reduced or abolished consciousness, such as sleep, pharmacological sedation and vegetative state and are likely contributing to overall alertness and attention (see Cavanna and Trimble, 2006, for ref.). Accordingly, the precuneus regions enlightened in the present study changes are activated during many sensorimotor and cognitive operations, such as eye movements (Simon et al., 2002), pointing (Astafiev et al., 2003), planning of finger movements (Jankowski et al., 2009), orofacial movement execution and learning (Arima et al., 2011), covert shift of attention to relevant spatial locations (Nagahama et al., 1999; Beauchamp et al., 2001), action observation and execution of observed actions (Chaminade et al., 2005), motor imagery (Ogiso et al., 2000; Malouin et al., 2003), visual memory tasks (Suchan et al., 2002), rhythm generation (Stevens et al., 2007). We may propose that, during malocclusion the performance of a sequence of finger displacements requires a higher attentive effort, possibly related to the spatial shift of the moving fingers and to the development of their visual image.

Significant clusters were also found in the posterior cingulate cortex on the right side. This region belong to DMF and is strongly coupled to the precuneus: its level of activation seems to increase with the level of arousal (Leech and Sharp, 2014). It has been proposed that it is involved in internally directed mental activity, in controlling the balance between internal and external attention and in the detection of environmental changes. The more simple explanation of the higher posterior cingulate activation during movement in Bite OFF is that this condition requires a higher mental and attentive effort. In effect, the region corresponding to the significant cluster in the posterior cingulate region has been also implicated in rhythm generation (Stevens et al., 2007), motor imagery (Malouin et al., 2003), planning of finger movements (Jankowski et al., 2009) and eye movements (Alvarez et al., 2010).

### Final Considerations

Why should a trigeminal unbalance lead to a cortical activation pattern indicating a larger effort in task performance and in the associated processes? A possibility is that the asymmetric discharge of muscle spindles, periodontal and, possibly, TMJ receptors induced by malocclusion leads to an unbalance in hemispheric excitability. There is indeed evidence that a hemispheric unbalance may deteriorate neural functions (Lomber and Payne, 1996). Moreover, unilateral stimulation of sensory afferents may relapse the symptoms induced by asymmetric brain lesions (Vallar et al., 1993). What could be the pathways involved in the postulated tonic control of trigeminal afferents on brain excitability? Neurons in the mesencephalic trigeminal nucleus are chemically (Luo et al., 1991) and, possibly, electrically (Fujita et al., 2012) coupled with the noradrenergic locus coeruleus (LC) neurons, which project to the whole brain (Samuels and Szabadi, 2008), controlling sensorimotor and cognitive processes (Berridge and Waterhouse, 2003). So, an unbalance in trigeminal afferents may leads to an unbalance in LC activity and, as a consequence, in brain excitability, since LC projections show an ipsilateral dominance. According to this hypothesis, it has been documented that trigeminal unbalance associated to malocclusion leads to an asymmetry in pupil size (De Cicco et al., 2014, 2016), which is a reliable indicator of LC discharge (Kihara et al., 2015), while occlusal correction reduces both trigeminal and pupil size asymmetry (De Cicco et al., 2014, 2016) and boost performance in complex sensorimotor tasks (De Cicco et al., 2016).

We must acknowledge that these results were obtained on a limited number of subjects and this can lower the statistical power of the study (Costafreda, 2009) and prevent a further generalization to a random population. A low statistical power increases the risk of a type II error and lowers the ability of discriminating whether non-significant results are due to a true absence of the hypothesized effect or to a limited number of subjects with respect to their standard deviation (Mumford, 2012). However, the occurrence of significant differences within several brain regions between Bite ON and OFF conditions in spite of the small number of subjects analyzed, suggests that the occlusal condition modulates brain activation elicited by skilled fingers movements and prompt the use of the Bite ON/Bite OFF paradigm for further investigate the issue in a larger population.

## Ethics Statement

This study was carried out in accordance with the recommendations of the Ethical Committee of the University of Pisa with written informed consent from all subjects. All subjects gave written informed consent in accordance with the Declaration of Helsinki. The protocol was approved by the Ethical Committee of the University of Pisa.

## Author Contributions

MPTF screened available databanks of brain activation and wrote parts of results and discussion. SD performed analysis of fMRI data, providing the task-rest BOLD signal maps, writing the Methods section and contributing to results discussion. VDC ideated the project, performed the odonthoiatric evaluations and corrections and contributed to results discussion. DM supervised the experiments and wrote parts of results and discussion. CT performed fMRI signals acquisition and participated in writing the Methods and discussing results. BC, DC, and GR contribute to odonthoiatric evaluations and corrections. MB contributed to results discussion and writing the paper. CV contributed to results discussion. UF revised the paper, made major changes to data analysis, interpretation and discussion.

## Conflict of Interest Statement

The authors declare that the research was conducted in the absence of any commercial or financial relationships that could be construed as a potential conflict of interest.

## Footnotes

1 http://www.fmrib.ox.ac.uk/fsl

2 http://brainmap.org/sleuth

3 http://neurosynth.org

4 http://sprout022.sprout.yale.edu/mni2tal/mni2tal.html
